# Histological and Immunohistochemical Characteristics of Mechanically Processed Adipose Tissue: A Systematic Review and Meta-Analysis

**DOI:** 10.3390/cells14211664

**Published:** 2025-10-23

**Authors:** Tom Schimanski, Rafael Loucas, Marios Loucas, Vanessa Brébant, Alexandra Anker, Silvan Klein, Sophia Theresa Diesch, Andrea Pagani, Lukas Prantl

**Affiliations:** 1 Department of Plastic, Hand and Reconstructive Surgery, University Hospital Regensburg, 93053 Regensburg, Germany; tom.schimanski@stud.uni-regensburg.de (T.S.); vanessa.brebant@ukr.de (V.B.); alexandra.anker@ukr.de (A.A.); silvan.klein@ukr.de (S.K.); sophia.diesch@ukr.de (S.T.D.); andrea.pagani@ukr.de (A.P.); lukas.prantl@ukr.de (L.P.); 2 Clinic of Plastic, Aesthetic, and Reconstructive Surgery, Döbling Private Hospital, 1090 Vienna, Austria; marios.loucas@hotmail.com

**Keywords:** adipose tissue, histology, immunohistochemistry, fat grafting, stromal vascular fraction, extracellular matrix, mechanical processing

## Abstract

Background: Mechanical processing techniques are commonly employed to prepare adipose tissue for clinical applications in reconstructive and aesthetic procedures. However, their histological and immunohistochemical impact on adipose tissue remains incompletely characterized. Purpose: This systematic review aims to investigate the impact of mechanical processing on the histological and immunohistochemical properties of adipose tissue. Methods: A systematic search was conducted using PubMed, Ovid, and Cochrane Library databases, with publications up to December 2024, employing Boolean operators (“mechanically processed” OR “lipoaspirate” OR “fat graft” OR “gauze rolling” OR “decantation” OR “coleman fat” OR “celt” OR “nanofat” OR “lipofilling” OR “human fat”) AND (“histol*”). Included were English-language studies or studies with a recognized English translation which had been subject to peer review and reported quantitative or qualitative markers of mechanically processed human adipose tissue with histology or immunohistochemistry. Risk of Bias was assessed with the OHAT score. Results: A total of 15 studies (*n* = 15) were included. In 13 of 15 studies (87%), mechanically processed adipose tissue demonstrated an increased stromal vascular fraction (SVF) cell density compared to unprocessed fat. Twelve studies (80%) reported improved preservation of the extracellular matrix (ECM), while 11 studies (73%) observed a reduction in mature adipocytes. Immunohistochemical analyses in 10 studies (67%) revealed elevated expression of vascular markers (CD31, CD34) and perilipin. Adverse histological features such as oil cysts, fibrosis, and inflammatory infiltrates were reduced in 9 studies (60%). Considerable heterogeneity in processing techniques and staining protocols precluded meta-analysis. Conclusions: Mechanical processing of adipose tissue is associated with favorable histological and immunohistochemical profiles, including increased SVF cell density, improved ECM preservation, and reduced inflammatory and fibrotic features. These findings support the potential of mechanical processing to enhance graft quality; however, standardization of techniques and evaluation protocols is needed to strengthen clinical translation.

## 1. Introduction

The usage of human fat in reconstructive surgery is not a recent development. Rather, it is a well-established and scientifically validated technique employed to address volume deficiencies and body contouring [1,2,3]. The utilization of fat tissue in regenerative medicine represents a relatively recent development. In this domain, adipocytes are injected into arthritic joints, such as the glenohumeral joint, the knee, and the small joints in the hand, with the objective of controlling inflammation and providing a lubricating effect [4,5,6]. It is a common practice to administer fat injections for the purpose of enhancing the aesthetic appearance and functional capabilities of the body. However, in certain instances, the adipose tissue proves to be too fragile, or alternatively, contains an insufficient quantity of stem cells. In the context of facial rejuvenation, it is imperative that the injected fat is resistant to shear force. It is of the utmost importance that the injected fat does not rupture during the injection process, particularly when using small cannulas. Strong facial impressions or mimicry can result into bursting of mature adipocytes (MAs) and therefore the release of free oil, which, in turn, can lead to the formation of oil cysts and local inflammations, thereby compromising graft survival [7]. In the context of joint injections, stem cells have been demonstrated to play a significant role in the protection of cartilage and the mitigation of inflammation [8,9]. To obtain the two characteristics under discussion, namely, increased stem cell density and increased susceptibility to shear force, it is crucial to eliminate the large MAs that are filled with oil [10,11].

The most common method for achieving this is through the application of enzymes or mechanical processing of the fat tissue. As demonstrated in the literature, there is a broad range of mechanical processing options available [10,12,13,14,15,16,17,18,19,20,21,22,23,24,25]. Therefore, in general, the application of mechanical forces is used to increase the concentration of the stromal vascular fraction. This is achieved by the elimination of lipid filled mature adipocytes, which are susceptible to shear forces [11]. Recent trends in clinical practice indicate a shift towards mechanical processing due to its relatively unregulated nature. Enzymes employed in the context of digestion must undergo rigorous cleansing prior to clinical utilization to not harm this surrounding tissue. Consequently, a straightforward yet efficacious mechanical processing protocol can be readily replicated within clinical environments [26,27].

A plethora of methodologies exist for the mechanical processing of fat tissue. These include the utilization of two luer-lock connected syringes, extracorporeal cutting devices, ultrasound, and commercial devices in conjunction with filtration and centrifugation to eliminate debris and impurities [28]. The difference between the injection of mechanically processed fat and untreated fat can be demonstrated through histological analysis. This can be done in two ways. Firstly, the stem cell density can be evaluated by analyzing samples taken directly after the processing. This is a way of validating the success of the process. Secondly, samples can be taken after explantation. This is a way of evaluating adverse clinical effects. These effects can include oily cysts, fibrosis, necrosis or inflammation [10,12,29].

The mechanical processing of human fat has been demonstrated to possibly result in the emulsification of fat tissue and fluids in the samples, thereby yielding a substandard product that exhibits a decrease in stem cell concentration [30]. Distinguishing this phenomenon is also possible through histological analysis, as demonstrated in the works of Cicione et al. (2023) and Eigenberger et al. (2025) [12,31]. The dissemination of histological images is a straightforward process, thus facilitating a more uniform comparison of various protocols for mechanical processing. The wide availability of this resource should also facilitate ease of access. With the assistance of trained pathologists, it is possible to acquire the necessary skills to evaluate histological slides in affordable time. As demonstrated in Schimanski et al. (2025), several standardized protocols have been developed for the evaluation of histological and immunohistochemistry images [29]. Presently, the majority of, but not all, researchers adhere to these established standards. The present systematic review has been undertaken with the objective of providing a comprehensive overview of the histological information obtained from slides of mechanically processed fat tissue, and of comparing this information with that obtained from slides of unprocessed fat tissue.

## 2. Methods

This systematic review was conducted in accordance with the PRISMA (Preferred Reporting Items for Systematic Reviews and Meta-Analyses) guidelines. The review protocol was prospectively registered with the Open Science Framework (Registration ID: osf.io/2yp7k).

### 2.1. Literature Search

A comprehensive search was performed in PubMed, Ovid, and the Cochrane Library for studies published up to December 2024. The following Boolean operators and keywords were used: (“mechanically processed” OR “lipoaspirate” OR “fat graft” OR “gauze rolling” OR “decantation” OR “Coleman fat” OR “CELT” OR “nanofat” OR “lipofilling” OR “human fat”) AND (“histol*”). The search aimed to identify all studies evaluating the histological and/or immunohistochemical characteristics of mechanically processed human adipose tissue. Only peer-reviewed articles published in English or with verified English translations were considered. No limitations were applied regarding publication year or study setting. In addition to database screening, the reference lists of included studies were manually reviewed to identify any additional eligible publications.

### 2.2. Eligibility Criteria

As can be seen in Table 1, studies were eligible for inclusion if they: Investigated human adipose tissue (in vitro or ex vivo). Employed mechanical processing methods (e.g., inter-syringe emulsification, filtration, decantation, gauze rolling). Included histological and/or immunohistochemical evaluation. Reported at least one qualitative or quantitative marker (e.g., stromal vascular fraction (SVF) density, adipocyte morphology, extracellular matrix (ECM) structure). Studies were excluded if they: Focused solely on enzymatic processing or isolated SVF cells without histological evaluation. Included only animal fat tissue. Were case reports, reviews, editorials, abstracts, or preclinical studies without original histological data. Duplicate datasets were identified by comparing author affiliations, sample characteristics, and study period; in such cases, only the most comprehensive publication was included.

### 2.3. Study Selection and Data Extraction

All search results were imported into Zotero 7.0.11 for reference management. Two independent reviewers screened titles and abstracts to identify potentially eligible studies. Full texts were then reviewed to confirm inclusion. Data were extracted using a standardized form, including: Study design and sample size, mechanical processing method, Histological or immunohistochemical technique, tissue attributes evaluated (e.g., adipocyte integrity, fibrosis, inflammation, vascular markers), main findings (qualitative and quantitative). Discrepancies between reviewers were resolved through discussion or consultation with a third reviewer.

### 2.4. Risk of Bias Assessment

The risk of bias for each included study was evaluated using the Office of Health Assessment and Translation (OHAT) Risk of Bias Rating Tool for Human and Animal Studies. This tool was selected for its suitability in assessing in vitro and ex vivo preclinical research, which comprised the majority of the included studies. Ten domains were assessed, with the exception of the criterion concerning toxicological exposure levels, which was not applicable in this context. Each domain was rated as: “++” (definitely low risk of bias), “+” (probably low risk), “−” (probably high risk), or “−−” (definitely high risk). Based on these ratings, studies were categorized into three levels: Level 1 (low risk of bias), if a maximum of one domain received a “−” rating and none received “−−”; Level 2 (moderate risk), if up to three domains were rated “−” and none “−−”; and Level 3 (high risk), if at least one domain was rated “−−” or more than three domains were rated “−”. Two independent reviewers conducted the assessment, and discrepancies were resolved through discussion to ensure consensus. For a detailed overview of the OHAT subcategory ratings across all studies, see Supplementary Figure S1.

### 2.5. Data Synthesis

Due to the heterogeneity in processing protocols, staining methods, and histological endpoints, a formal meta-analysis was not feasible. Instead, findings were synthesized descriptively and summarized in tabular format. Key outcomes included trends in SVF enrichment, ECM preservation, adipocyte fragmentation, and immunohistochemical marker expression.

### 2.6. Statistical Analysis

Due to substantial heterogeneity in study designs, mechanical processing protocols, histological endpoints, and outcome reporting, a meta-analytical synthesis was not performed. Instead, a qualitative descriptive synthesis was undertaken. Extracted data were summarized using frequencies and proportions to identify common histological and immunohistochemical outcomes across studies. These included changes in stromal vascular fraction (SVF) cell density, extracellular matrix (ECM) integrity, adipocyte morphology, and presence of fibrosis, inflammation, or oil cysts. Where applicable, semiquantitative and quantitative results were tabulated to facilitate cross-study comparison. Inferential analysis was not applicable due to heterogeneity.

## 3. Results

### 3.1. Study Selection

**Figure 1:**
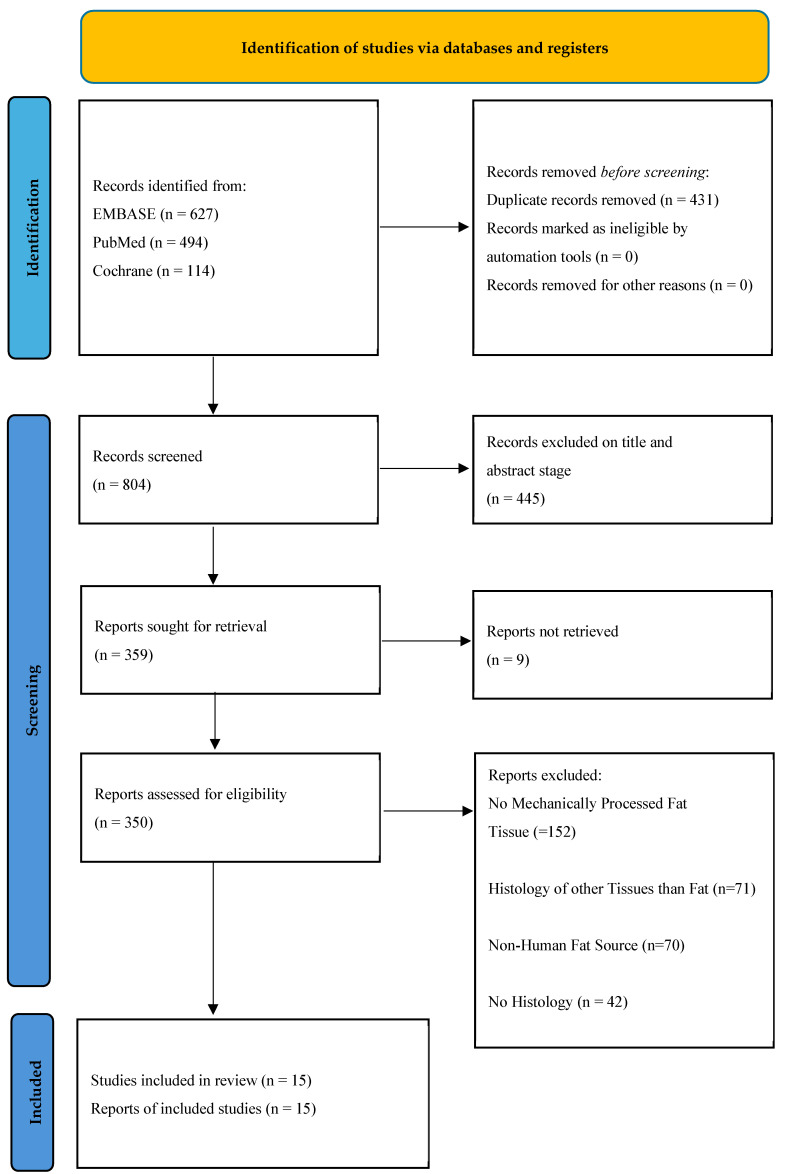
PRISMA 2020 flow diagram to identify the studies which fulfill the inclusion criteria [32,33,34].

A total of 1235 records were retrieved from database searches. After eliminating 431 duplicates, 804 articles remained for title and abstract screening. Of these, 359 full-text articles were reviewed for eligibility, leading to the exclusion of 344 studies. Ultimately, 15 studies met the inclusion criteria, all of which investigated histological and immunohistochemical assessments of mechanically processed human adipose tissue. A PRISMA flow diagram illustrating the study selection process is provided in Figure 1, and the concrete 15 included studies in Table 2. Their mechanical processing techniques are explained in Table 3.

### 3.2. Study Characteristics

A total of 15 studies were included in this systematic review. Of these, 5 (33%) were in vitro experimental studies using freshly harvested human adipose tissue. Nine studies (60%) were conducted in a preclinical setting using murine models, and one study (7%) was a clinical investigation evaluating explanted human fat following transplantation. The studies were published between 2013 and 2024. Sample sizes ranged from 1 to 11 patients per cohort, with a predominance of female participants. Patient demographics, including age, sex, and body mass index (BMI), were reported in 11 studies (73%). However, reporting of these parameters was inconsistent. In 11 out of 15 studies (73%), processed adipose tissue was directly compared to unprocessed tissue from the same individual, allowing for internal control. The number of adipose tissue samples analyzed per patient varied, as multiple replicates were frequently obtained from individual donors. No study was rated as having a high risk of bias; therefore, a sensitivity analysis was not deemed necessary.

### 3.3. Histological Staining Techniques

Standard histological staining was performed in all 15 studies (100%). Hematoxylin and eosin (H&E) was used in 14 studies (93%), making it the most commonly employed method for assessing tissue architecture, adipocyte morphology, and cellular distribution. Masson’s trichrome staining was used in 4 studies (27%) to evaluate collagen deposition and extracellular matrix (ECM) structure. Other staining techniques were used sporadically, and details of fixation, sectioning, and image analysis protocols varied across studies.

### 3.4. Immunohistochemical Analysis

**Figure 2:**
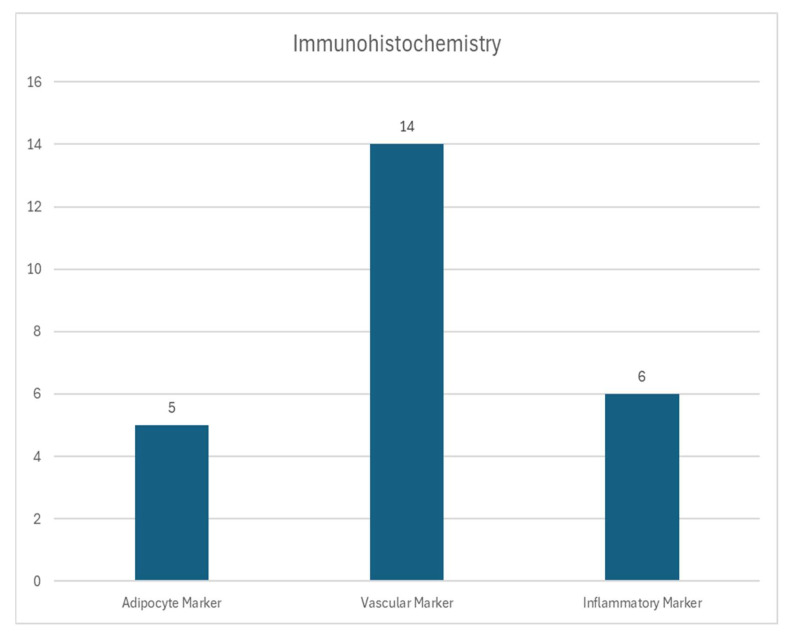
The graph illustrates the use of Antibodies for immunohistochemistry in the reviewed papers, which have been categorized into four groups. The following antibodies were detected: anti-adipocyte markers (perilipin); vascular markers (CD31, CD34, α-SMA, CD146, CD90 and PCNA); inflammatory markers (F4/80, CD206 and MAC).

Immunohistochemistry was conducted in 11 studies (73%). Vascular markers were the most frequently assessed, with CD31 used in 7 studies (47%) and CD34 in 5 studies (33%). Perilipin, used to detect mature adipocytes, was applied in 5 studies (33%). Other markers included α-SMA (used in 4 studies), CD90 (3 studies), CD146 (2 studies), and PCNA (2 studies), which were employed to identify proliferating cells, pericytes, and stromal components. Inflammatory markers were analyzed in 5 studies (33%), including F4/80 (4 studies), CD206 (2 studies), and MAC2 (2 studies). A visual summary of immunohistochemical markers used is presented in Figure 2.

### 3.5. In Vitro Protocols

Among the five in vitro studies (33%), no evaluation of adverse histological features such as oil cysts, necrosis, fibrosis, or inflammatory infiltrates was reported. Four studies (80%) used H&E staining, and two studies (40%) employed Masson’s trichrome. All studies conducted immunohistochemical analyses focusing on cellular composition. Frequently used markers included CD31 (3 studies), CD34 (3 studies), PCNA (2 studies), perilipin (2 studies), and α-SMA (2 studies). Macrophage presence was described in three studies, with MAC2 included in two protocols.

### 3.6. Preclinical/Clinical Protocols

**Figure 3:**
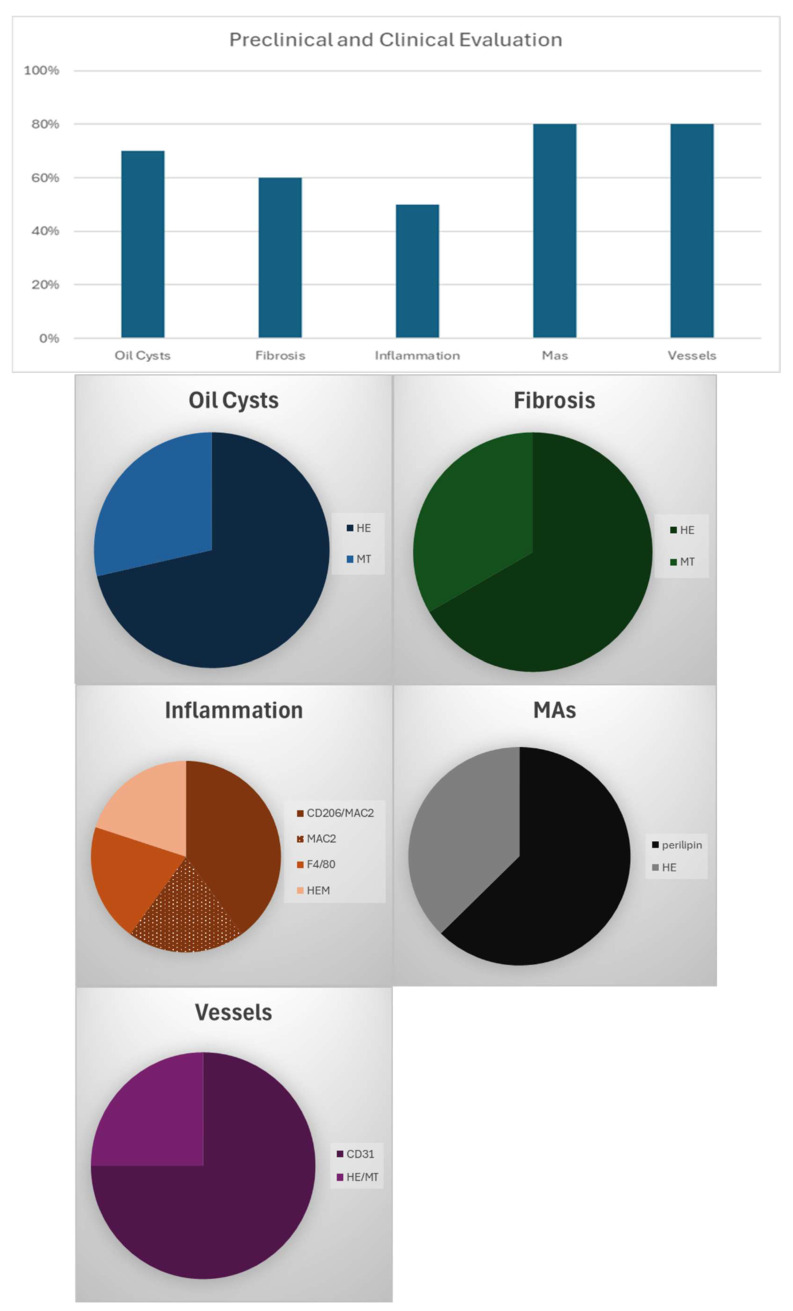
The graph illustrates the proportion of studies that have evaluated these attributes. The following series of circle diagrams provides a visual representation of the staining employed in the evaluation of specific attributes.

Ten studies (67%) included either preclinical or clinical evaluation of fat grafts following transplantation. Histological outcomes were reported across 30 tissue-specific assessments. Of these, 18 evaluations (60%) investigated features such as inflammation, fibrosis, or oil cysts, while 20 evaluations (67%) focused on adipocyte morphology or vascularization. Intact mature adipocytes and identifiable blood vessels were reported in 16 of these 20 assessments (80%). Immunohistochemical analysis in these studies used various combinations of CD31, CD34, α-SMA, perilipin, and inflammatory markers. Figure 3 presents the proportion of studies evaluating specific histological attributes and corresponding staining methods.

### 3.7. Mechanical Processing Techniques

All 15 studies (100%) described the mechanical processing protocols used for adipose tissue preparation. Methods included inter-syringe emulsification (*n* = 9), filtration (*n* = 6), gauze rolling (*n* = 3), decantation (*n* = 4), and proprietary device-based processing (*n* = 2). Descriptions typically included stepwise procedures, device specifications, processing duration, and applied forces or pressure gradients. However, terminology, procedural definitions, and reported parameters varied considerably, limiting reproducibility and standardization. A detailed overview of all protocols is presented in Table 3.

### 3.8. Methodological Variability

Substantial heterogeneity was noted across studies in terms of mechanical processing methods, sample preparation, histological and immunohistochemical staining, and data presentation. Only 4 of 15 studies (27%) utilized semiquantitative scoring or standardized histological grading systems. Due to the lack of uniform outcome definitions and comparable metrics, data pooling for meta-analysis was not feasible. All outcomes were therefore summarized descriptively.

## 4. Discussion

This systematic review is the only systematic review that evaluated the histological and immunohistochemical changes in adipose tissue following mechanical processing, across 15 studies including in vitro, preclinical, and one clinical investigation. In contrast to other reviews in this field, the present study concentrated more on the histological properties found in mechanically processed tissues. In contrast, Schipper et al. (2023) focused more on clinical outcomes, such as skin rejuvenation or wound healing [28]. However, this study provides a well-structured description of how histology is used in the literature to evaluate adipose tissue. Therefore, this build the foundation for the subsequent evaluation of the histological properties of fat tissue, with the goal to attain a comprehensive understanding of histology and immunohistochemistry and its utilization within the extant literature. Despite the growing interest in regenerative applications of fat grafting, considerable variability persists in processing protocols, tissue handling, and analytical methodologies.

Given the absence of a standardized nomenclature for mechanically processed fat, the following discussion aligns with the designated names used in the corresponding studies. The precise protocols are described in the abbreviation list in Table 2.

The most frequently employed mechanical processing technique included a combination of centrifugation and tissue disruption using a three-way stopcock. Notably, Cicione et al. [12], Tran et al. [18], and Yao et al. [21] applied identical centrifugation parameters—1200× *g* for 3 min—both before and after mechanical disruption. In contrast, Eigenberger et al. [10] utilized a shorter centrifugation time at higher g-forces, raising concerns regarding potential stromal vascular fraction cell (SVFC) damage. However, Pulsfort et al. [35] demonstrated that SVFCs can tolerate forces up to 20,000× *g* without compromising viability, suggesting that optimized high-speed centrifugation may aid in debris clearance. Some protocols, such as that of Zhao et al. [24], omitted the second centrifugation step, recommending instead that low-liquid, well-sedimented fat be used for disruption to avoid product emulsification. This concern was echoed by Eigenberger et al. [31], who linked emulsification to impaired SVFC yield. Similarly, van Dongen et al. [30] compared sedimentation versus centrifugation and concluded that the latter is essential for validating successful volume reduction and avoiding retained oil.

Filtration was included in five studies, but Ramaut et al. [36] reported a significant decrease in SVFC counts following this step, cautioning against filtration as it may counteract the goal of stromal enrichment. Several protocols utilized commercial mechanical devices [12,15,16,19], which are praised for ease of use but associated with higher per-use costs compared to standard luer-lock and stopcock methods.

Experimental in vitro studies focused primarily on the enrichment of stromal cells and reduction in mature adipocyte (MA) content. Cicione et al. [12] demonstrated that the three-way stopcock method outperformed the Lipogems system in both reducing MAs and increasing SVFCs. Ragni et al. [15] similarly utilized the Lipogems system and found its outcomes to be comparable to unprocessed lipoaspirate in terms of vessel density, though red blood cell content was markedly lower, suggesting a potential reduction in post-injection inflammation. Von Dongen et al. [30] emphasized both cost and processing efficiency and found a significant increase in small vessels and ECM, alongside reduced MAs, in total stromal vascular fraction (tSVF) compared to unprocessed fat. These findings were confirmed histologically using perilipin immunostaining.

Sesé et al. [17] used Masson trichrome staining with quantitative image analysis via ImageJ (unknown version) to demonstrate a substantial increase in collagen content post-processing. Eigenberger et al. [31] reported similar findings but extended the analysis to include MA counts, showing a significant reduction. They further described alterations in adipocyte size and distribution patterns across samples, suggesting that mechanical processing influences both the cellular and structural composition of fat.

In preclinical and clinical studies, attention shifted toward evaluating fibrosis, oil cyst formation, and inflammation in explanted grafts. Fan et al. [13] reported decreased fibrosis and higher capillary density in mechanically processed grafts and found that extracorporeal shredding methods significantly reduced surgical time. He et al. [14] found elevated levels of CD31 and Ki67 in ultrasound-processed fat, indicating increased vascularization and cellular proliferation. However, due to the absence of prior centrifugation, residual MAs were retained, which could explain the oil cyst formation observed during explantation.

Tran et al. [18] and Yu et al. [22] implemented a semi-quantitative scoring system, also validated by Schimanski et al. [29], to grade histological features such as fibrosis, inflammation, and oil cysts on a 1–5 scale. Tran et al. [18] found that centrifuged samples exhibited less inflammation and fewer oil cysts than either filtered or unprocessed samples. Yao et al. [21] further confirmed the superiority of mechanically processed fat across all histological categories.

To characterize inflammatory responses, Wu et al. and Zhang et al. [20,23] utilized CD206 for M1 (pro-inflammatory) and MAC2 for M2 (anti-inflammatory) macrophages subtypes. It is imperative to acknowledge that the distinction between M1 and M2 macrophages is characterized by the use of different markers across different studies, which may exhibit discrepancies when comparing them [37]. This should be considered during study design or interpretation of studies. Wu et al. [20] reported increased angiogenesis and adipogenesis in mechanically processed fat, attributing this to a favorable shift in macrophage populations. Zhang et al. [23] also performed immunostaining for HLA and found that new adipocytes lacked HLA expression, suggesting a non-human (murine) origin in xenografts, a novel observation with important implications for fat graft biology.

Säljö et al. [16] noted rapid stem cell dissolution following implantation, coinciding with intense neovascularization. Yao et al. [21] was the only author to conduct a human study, reporting a structurally intact fat graft without calcifications or oil cysts. Zhu et al. [25] showed that low-density fat, derived from the upper phase after centrifugation, had reduced SVFC content but, after mechanical processing, yielded enhanced histological results including less fibrosis, fewer oil cysts, and greater mature adipocyte formation.

Taken together, the included studies demonstrate that mechanical processing significantly alters the histological profile of adipose tissue by enriching stromal elements, preserving ECM structure, and reducing mature adipocyte content. All studies that evaluated MAs and vessels via immunohistochemistry found a reduction in intact MAs after processing, as well as denser vessels. These tissue-level changes appear to support regenerative applications; however, methodological heterogeneity remains a central challenge.

In the context of research involving mechanically isolated SVF cells, it is imperative to consider enzymatically isolated SVF cells as well, given the documented advantages and disadvantages inherent to both methodologies, as evidenced by Uguten et al. (2024) [26]. A comparison of mechanically processed fat tissue and enzymatically isolated stromal vascular fraction (SVF) cells reveals several notable differences. When performed correctly, the enzymatical isolation yields a more concentrated product devoid of impurities. However, the absence of a scaffolding component, the extracellular matrix (ECM), renders the procedure ineffective for addressing volume deficiencies. In addition, it is imperative that collagenase be administered only after a thorough rinse and elimination process to avoid harmful effects on the tissue [38]. Integration of enzymatically treated fat tissue into the present review would result in a scattered scope, necessitating careful consideration to ensure the integrity of the review’s findings.

Most notably, there was wide variability in processing techniques (centrifugation forces, filtration steps, emulsification methods), in staining protocols and outcome definitions as well as validation of antibodies for immunohistochemistry. While hematoxylin and eosin staining was nearly universal, only a few studies applied validated scoring systems or quantitative digital tools. Furthermore, the majority of studies lacked long-term in vivo follow-up, and very few linked histological changes to functional outcomes such as graft retention, inflammation, or patient satisfaction. The number of included studies is relatively small, which limits the generalizability of the findings. In addition, publication bias was transparently assessed; however, its existence cannot be ruled out, as studies with positive findings are more likely to be published. The utilization of disparate stains and antibodies engenders considerable complexity in direct comparability. A final consideration is the potential for an overlap in authorship and geopolitical clustering of datasets to exert an influence on the synthesis. This factor must be considered during the interpretation of the results.

The presence of a multitude of protocols for mechanical processing within the extant literature is indicative of the prevailing uncertainty surrounding the optimal parameters for producing a product that is both high-quality and reproducible. To ascertain the most effective protocol, it is necessary to undertake a rigorous and systematic investigation. In the context of future research, this should be a central focus. The resolution may be achieved through a comparative analysis of different setups varying in the used parameters as source and strength of shear force and centrifugation forces, plus the automatization of the process itself.

Despite these limitations, the histological and immunohistochemical findings consistently support the hypothesis that mechanical processing enhances the regenerative quality of adipose tissue. Further studies are warranted to establish standardized protocols and to correlate histological observations with clinical outcomes, especially in human models.

## 5. Conclusions

Mechanical processing of adipose tissue consistently alters its histological and immunohistochemical properties, resulting in increased stromal vascular fraction cell content, preserved extracellular matrix architecture, and a reduction in mature adipocytes. These modifications may enhance the regenerative potential of fat grafts. Despite consistent findings across multiple studies, significant methodological heterogeneity in processing techniques and evaluation protocols limits direct comparability and clinical translation. Standardized protocols and outcome measures are needed to better define the role of mechanically processed adipose tissue in regenerative medicine and to facilitate integration into clinical practice.

## Figures and Tables

**Table 1:** Inclusion and Exclusion Criteria.

Criteria	Inclusion	Exclusion
Study Type	Preclinical and experimental studies with histology of fat tissue which underwent mechanical processing	Case reports, editorials, commentaries, reviews without original data, systematic reviews or meta-analyses
Population	All human studies	All animal studies
Intervention	Whole tissue	Studies focusing only on stem cells of the fat (SVF) and their histology
Publication Date	All studies available (2013–2024)	-
Language	Published in English	Non-English language studies
Peer-reviewed	Studies must be published in peer-reviewed journals	Non-peer-reviewed articles

**Table 2:** Data of the 15 included Studies.

Study Author	Year	OHAT Score	Study Design	Cohort Size	Number of Samples		Time of Explantation	Staining Method
					Processed	Not Processed		
Cicione et al. [12]	2023	Level 1	Experimental	9♀, 9♂ 63 ± 11 y	6 MAT, 6 NAT	6 LA	-	HE, Anti-PCNA, Anti-CD31, Anti-CD34, Anti-COLL1
Eigenberger et al. [10]	2022	Level 1	Experimental	11♀; 25–62 y; BMI: 29.6 ± 5.2 kg/m^2^	11 CELT; 11 CELT^PLUS^	11 S	-	HE
Fan et al. [13]	2021	Level 1	preclinical experimental	♀; 20–55Y; BMI: 21–31 kg/m^2^	10 AERF	10 CF	10 weeks	HE, Anti-CD31, Anti-perilipin
He et al. [14]	2023	Level 2	Preclinical experimental	6♀; 20–30 y	6 NF; 6 CUPF	6 MF; 6 C	4 weeks	HE, Anti-Ki67, Anti-CD31
Ragni et al. [15]	2022	Level 1	Experimental	4♀, 3♂ 44 ± 6 y	-	-	-	HE, Anti-CD31, Anti-CD90, Anti-CD146
Säljö et al. [16]	2020	Level 2	Preclinical experimental	2♀	9 LAT		3, 7, or 30 days	HE, Anti-CD31, Anti-CD90
Sesé et al. [17]	2020	Level 1	Experimental	6♀; 18–45 y; BMI: 21.5 ± 2 kg/m^2^	6 SCA	6 LA	-	HE, Masson Trichrome
Tran et al. [18]	2024	Level 1	Preclinical experimental	1♀; 47 y	5 NF; 5 LC	5 CF	4 weeks	HE, Anti-CD31, Anti-F4/80, Anti-perilipin
Van Dongen et al. [19]	2020	Level 2	Experimental	3	3 tSVF	3 LA	-	Masson Trichrome Anti-perilipin, Anti-αSMA
Wu et al. [20]	2023	Level 1	Preclinical experimental	16–60 y BMI: 18.3–31.5 kg/m^2^	24 UCF	24 CF	3, 7, 14, 30, 60, or 90 days	HE, Anti-CD31, Anti-perilipin, Anti-galectin 3, Anti-CD206
Yao et al. [21]	2013	Level 2	Clinical experimental	92♀, 34♂ 34.7 ± 9.3 y BMI: 23.4 ± 2.1 kg/m^2^	1 SFVG	-	12 months	HE
Yu et al. [22]	2018	Level 1	Preclinical experimental	5♀; 25–38 y	10 Nano100; 10 Nano200	10 MF	12 weeks	HE, Masson Trichrome, Anti-CD31
Zhang et al. [23]	2018	Level 1	Preclinical experimental	11♀; 33.4 ± 6.3 y; BMI: 23.2 ± 1.9 kg/m^2^	63 SVFG	63 CF	1, 3, 5, 7, 11, 15, 30, 60, or 90 days	HE, Masson Trichrome, Anti-perilipin, Anti-Mac2, Anti-CD206, Anti-HLA
Zhao et al. [24]	2023	Level 2	Preclinical experimental	-	2 SVFG	-	14 and 28 days	HE
Zhu et al. [25]	2021	Level 1	Preclinical experimental	8♀	6 CLDF	6 CF, 6 HDF, 6 LDF	3 months	HE, Anti-Mac2, Anti-perilipin,

♀: female; ♂: male; y: Years of age; BMI: body mass index; MAT: micro-fragmented adipose tissue; NAT: nanofat adipose tissue; LA: lipoaspirate; CELT: cleaned and enriched lipid tissue; CELT^PLUS^: cleaned and enriched lipid tissue—purified long-lasting ultra-concentrated supergraft; S: sedimented; AERF: adipose-derived progenitor cell enrichment fat; NF: Nanofat; CUPF: concentrated ultrasound-processed fat; MF: microfat; C: cetrifugate; LAT: lipoaspirate-derived adipose tissue; SCA: stromal cell agregartes; tSVF: tissue stromal vascular fraction; LC: lipoconcentrate; UCF: Ultra-condensed fat; CF: Coleman fat; SVFG: stromal vascular fraction gel; Nano100: 9% nanofat; Nano200: 17% nanofat; CLDF: condensed low-density fat; HDF: high-density fat; LDF: low-density fat.

**Table 3:** Mechanical processing maneuvers used in the 15 Studies.

Study Author	Protocol Maneuver
Cicione et al. [12]	MAT: Lipogems© processing kit; NAT: C: 1200× *g* 3 min; emulsification with 90° stopcock; C: 1200× *g* 3 min
Eigenberger et al. [10]	CELT: C: 1600× *g* 2 min CELT PLUS: C: 1600× *g* 2 min; emulsification with 90° stopcock; C: 1600× *g* 2 min
Fan et al. [13]	AERF: Extracorporal cutting device with 1500–1600 rpm; F: 100 mesh
He et al. [14]	NF: rinsed; emulsified with Luer-Lock connector; C: 300× *g* 3 min CUPF: ultrasonic disruption for 15–20 s and 30 W; C: 1200× *g* 5 min
Ragni et al. [15]	µFAT: Lipogems© processing kit 120 TM and 240 TM
Säljö et al. [16]	LAT: Lipogems© processing kit
Sesé et al. [17]	SCA: C: 1200× *g* 3 min; emulsification with 90° stopcock; C: 800× *g* 10 min
Tran et al. [18]	NF: F: Nylon cloth 0.5 mm pores; emulsification with 2.4 mm and 1.2 mm connector; F: Nylon cloth 0.5 mm pores LC: C: 1200× *g* 3 min; emulsification with 2.4-mm and 1.2-mm connector; C: 1200× *g* 3 min
Van Dongen et al. [19]	tSVF: 1-hole and 3-hole fractionator Tulip Medical Products with centrifugation before and after
Wu et al. [20]	UCF: C: 1200× *g* 3 min; Agitate and Rest 5 min
Yao et al. [21]	SVFG: C: 1200× *g* 3 min; emulsification with stopcock; C: 1200× *g* 3 min
Yu et al. [22]	NF: F: Nylon cloth 0.5 mm pores; emulsification with female-to-female Luer-Lock; F: Nylon cloth 0.5-mm pores
Zhang et al. [23]	SVFG: C: 1200× *g* 3 min; emulsification with stopcock; F: 500 mym; C: 2000× *g* 3 min
Zhao et al. [24]	SVFG: emulsification with stopcock; C: 2000× *g* 3 min
Zhu et al. [25]	CLDF: C: 1200× *g* 3 min; upper half emulsified with female-to-female Luer-Lock; C: 1600× *g* 3 min

C: centrifugation; F: filtrated.

## Data Availability

Data is available in Table 1 and Table 2 and towards request to our Authors.

## Supplementary Materials

The following supporting information can be downloaded at: https://www.mdpi.com/article/10.3390/cells14211664/s1.

## References

[1] Guerrerosantos J. (1996). Autologous fat grafting for body contouring. Clin. Plast. Surg..

[2] Shibahara T., Watanabe Y., Yamaguchi S., Noma H., Yamane G.Y., Abe S., Ide Y. (1996). Use of the buccal fat pad as a pedicle graft. Bull. Tokyo Dent. Coll..

[3] Drommer R.B., Mende U., Krifka F.J. (1995). Die freie Fetttransplantation im Gesichtsbereich. Hautarzt.

[4] Natali S., Screpis D., Patania E., De Berardinis L., Benoni A., Piovan G., Iacono V., Magnan B., Gigante A.P., Zorzi C. (2023). Efficacy and Long-Term Outcomes of Intra-Articular Autologous Micro-Fragmented Adipose Tissue in Individuals with Glenohumeral Osteoarthritis: A 36-Month Follow-Up Study. J. Pers. Med..

[5] Froschauer S.M., Holzbauer M., Wenny R., Schmidt M., Huemer G.M., Kwasny O., Duscher D. (2020). Autologous Fat Transplantation for Thumb Carpometacarpal Joint Osteoarthritis (Liparthroplasty): A Case Series with Two Years of Follow-UP. J. Clin. Med..

[6] Weninger P., Feichtinger X., Steffel C., Kerschbaumer C., Duscher D. (2023). Arthroscopy with Lipoaspirate and Plasma Infiltration Using Adipose-Derived Stem Cells Plus Platelet-Rich Plasma: Harvesting and Injection for Arthroscopic Treatment of Cartilage Defects of the Knee. Arthrosc. Tech..

[7] Prantl L., Brix E., Kempa S., Felthaus O., Eigenberger A., Brébant V., Anker A., Strauss C. (2021). Facial Rejuvenation with Concentrated Lipograft—A 12 Month Follow-Up Study. Cells.

[8] Onorato F., Rucci M., Alessio-Mazzola M., Bistolfi A., Castagnoli C., Formica M., Ferracini R. (2024). Autologous microfragmented adipose tissue treatment of knee osteoarthritis demonstrates effectiveness in 68% of patients at 4-year follow-up. Arch. Orthop. Trauma Surg..

[9] Yu Y., Lu Q., Li S., Liu M., Sun H., Li L., Han K., Liu P. (2023). Intra-Articular Injection of Autologous Micro-Fragmented Adipose Tissue for the Treatment of Knee Osteoarthritis: A Prospective Interventional Study. J. Pers. Med..

[10] Eigenberger A., Felthaus O., Schratzenstaller T., Haerteis S., Utpatel K., Prantl L. (2022). The Effects of Shear Force-Based Processing of Lipoaspirates on White Adipose Tissue and the Differentiation Potential of Adipose Derived Stem Cells. Cells.

[11] Prantl L., Eigenberger A., Klein S., Limm K., Oefner P.J., Schratzenstaller T., Felthaus O. (2020). Shear Force Processing of Lipoaspirates for Stem Cell Enrichment Does Not Affect Secretome of Human Cells Detected by Mass Spectrometry In Vitro. Plast. Reconstr. Surg..

[12] Cicione C., Vadalà G., Di Giacomo G., Tilotta V., Ambrosio L., Russo F., Zampogna B., Cannata F., Papalia R., Denaro V. (2023). Micro-fragmented and nanofat adipose tissue derivatives: In vitro qualitative and quantitative analysis. Front. Bioeng. Biotechnol..

[13] Fan P., Fang M., Li J., Solari M.G., Wu D., Tan W., Wang Y., Yang X., Lei S. (2021). A Novel Fat Making Strategy With Adipose-Derived Progenitor Cell-Enriched Fat Improves Fat Graft Survival. Aesthet. Surg. J..

[14] He J., Chen Fzhou Zhang Y., Tan Pching Li Q., Cheng C. (2023). Concentrated ultrasound-processed fat (CUPF): More than a mechanically emulsified graft. J. Plast. Reconstr. Aesthet. Surg..

[15] Ragni E., Viganò M., Torretta E., Perucca Orfei C., Colombini A., Tremolada C., Gelfi C., de Girolamo L. (2022). Characterization of Microfragmented Adipose Tissue Architecture, Mesenchymal Stromal Cell Content and Release of Paracrine Mediators. J. Clin. Med..

[16] Säljö K., Orrhult L.S., Apelgren P., Markstedt K., Kölby L., Gatenholm P. (2020). Successful engraftment, vascularization, and In vivo survival of 3D-bioprinted human lipoaspirate-derived adipose tissue. Bioprinting.

[17] Sesé B., Sanmartín J.M., Ortega B., Llull R. (2020). Human Stromal Cell Aggregates Concentrate Adipose Tissue Constitutive Cell Population by In Vitro DNA Quantification Analysis. Plast. Reconstr. Surg..

[18] Tran V.V.T., Hong K.Y., Jin X., Chang H. (2024). Histological Comparison of Nanofat and Lipoconcentrate: Enhanced Effects of Lipoconcentrate on Adipogenesis and Angiogenesis. Aesthetic Plast. Surg..

[19] Van Dongen J.A., Gostelie O.F.E., Vonk L.A., De Bruijn J.J., Van Der Lei B., Harmsen M.C., Stevens H.P. (2020). Fractionation of Adipose Tissue Procedure With a Disposable One-Hole Fractionator. Aesthet. Surg. J..

[20] Wu W., Bi X., Zhao J., Lin Z., Lu F., Dong Z., Li Y. (2023). Ultra-condensed Fat: A Novel Fat Product for Volume Augmentation. Aesthetic Plast. Surg..

[21] Yao Y., Cai J., Zhang P., Liao Y., Yuan Y., Dong Z., Lu F. (2018). Adipose Stromal Vascular Fraction Gel Grafting: A New Method for Tissue Volumization and Rejuvenation. Dermatol. Surg..

[22] Yu Q., Cai Y., Huang H., Wang Z., Xu P., Wang X., Zhang L., Zhang W., Li W. (2018). Co-Transplantation of Nanofat Enhances Neovascularization and Fat Graft Survival in Nude Mice. Aesthet. Surg. J..

[23] Zhang Y., Cai J., Zhou T., Yao Y., Dong Z., Lu F. (2018). Improved Long-Term Volume Retention of Stromal Vascular Fraction Gel Grafting with Enhanced Angiogenesis and Adipogenesis. Plast. Reconstr. Surg..

[24] Zhao P., Wang B., Wang L., Fu Z., Hu J., Liu Y., Wang J., He Y. (2023). Rapid printing of 3D porous scaffolds for breast reconstruction. Bio-Des. Manuf..

[25] Zhu H., Quan Y., Wang J., Jiang S., Lu F., Cai J., Liao Y.M. (2021). Improving Low-Density Fat by Condensing Cellular and Collagen Content through a Mechanical Process: Basic Research and Clinical Applications. Plast. Reconstr. Surg..

[26] Uguten M., van der Sluis N., Vriend L., Coert J.H., Harmsen M.C., van der Lei B., van Dongen J.A. (2024). Comparing mechanical and enzymatic isolation procedures to isolate adipose-derived stromal vascular fraction: A systematic review. Wound Repair. Regen..

[27] Aronowitz J.A., Lockhart R.A., Hakakian C.S. (2015). Mechanical versus enzymatic isolation of stromal vascular fraction cells from adipose tissue. Springerplus.

[28] Schipper J., Van Laarhoven C., Schepers R., Tuin A., Harmsen M., Spijkervet F., Jansma J., van Dongen J.A. (2023). Mechanical Fractionation of Adipose Tissue—A Scoping Review of Procedures to Obtain Stromal Vascular Fraction. Bioengineering.

[29] Schimanski T., Loucas R., Loucas M., Felthaus O., Brébant V., Klein S., Anker A., Frank K., Siegmund A., Pagani A. (2025). Histology and Immunohistochemistry of Adipose Tissue: A Scoping Review on Staining Methods and Their Informative Value. Cells.

[30] Van Dongen J.A., Tuin A.J., Harmsen M.C., Van Der Lei B., Stevens H.P. (2020). The Difference between Stromal Vascular Fraction Isolation and Fat Emulsification: A Crucial Role for Centrifugation. Plast. Reconstr. Surg..

[31] Eigenberger A., Felthaus O., Bartsch A., Schimanski T., Utpatel K., Prantl L. (2025). The Influence of Sedimentation on the Composition of the Lipoaspirate and the Effects on Further Mechanical Processing. Cells.

[32] Howick J., Chalmers I., Glasziou P., Greenhalgh T., Heneghan C., Liberati A., Moschetti I. (2011). The 2011 Oxford CEBM Levels of Evidence (Introductory Document). Oxford Centre for Evidence-Based Medicine. https://www.cebm.ox.ac.uk/resources/levels-of-evidence/ocebm-levels-of-evidence.

[33] Paiva Barbosa V., Bastos Silveira B., Amorim Dos Santos J., Monteiro M.M., Coletta R.D., De Luca Canto G., Stefani C.M., Guerra E.N.S. (2023). Critical appraisal tools used in systematic reviews of in vitro cell culture studies: A methodological study. Res. Synth. Methods.

[34] Page M.J., McKenzie J.E., Bossuyt P.M., Boutron I., Hoffmann T.C., Mulrow C.D., Shamseer L., Tetzlaff J., Akl E., Brennan S.E. (2021). The PRISMA 2020 statement: An updated guideline for reporting systematic reviews. BMJ.

[35] Pulsfort A.K., Wolter T.P., Pallua N. (2011). The Effect of Centrifugal Forces on Viability of Adipocytes in Centrifuged Lipoaspirates. Ann. Plast. Surg..

[36] Ramaut L., Moonen L., Laeremans T., Aerts J.L., Geeroms M., Hamdi M. (2023). Push-Through Filtration of Emulsified Adipose Tissue Over a 500-µm Mesh Significantly Reduces the Amount of Stromal Vascular Fraction and Mesenchymal Stem Cells. Aesthet. Surg. J..

[37] Martinez F.O., Gordon S. (2014). The M1 and M2 paradigm of macrophage activation: Time for reassessment. F1000Prime Rep..

[38] Zinger G., Gronovich Y., Lotan A.M., Sharon-Gabbay R. (2024). Pilot Study for Isolation of Stromal Vascular Fraction with Collagenase Using an Automated Processing System. Int. J. Mol. Sci..

