# Supplementary material for: Histological and Immunohistochemical Characteristics of Mechanically Processed Adipose Tissue: A Systematic Review and Meta-Analysis
Source: Cells. 2025 Oct 23;14(21):1664. doi: 10.3390/cells14211664 (PMC12608007; doi:10.3390/cells14211664)
Supplement: Supplementary file 1 [file cells-14-01664-s001.zip › cells-3936225-supplementary.pdf]

Histological and Immunohistochemical Characteristics of Mechanically Processed Adipose Tissue: A Systematic Review and Meta-Analysis

Tom Schimanski [1,†], Rafael Loucas [1,*,†], Marios Loucas [2], Vanessa Brébant [1], Alexandra Anker [1], Silvan Klein [1], Sophia Theresa Diesch [1], Andrea Pagani [1] and Lukas Prantl [1]

1 Department of Plastic, Hand and Reconstructive Surgery, University Hospital Regensburg, 93053 Regensburg, Germany

2 Clinic of Plastic, Aesthetic, and Reconstructive Surgery, Döbling Private Hospital, 1090 Vienna, Austria

† These authors contributed equally to the study

Corresponding Author

Rafael Loucas, MD

Department of Plastic, Hand and Reconstructive Surgery, University Hospital Regensburg

Franz-Josef-Strauss-Allee 11

93053 Regensburg

Germany

Financial Disclosure Statement:

The authors have no financial interest in any of the products, devices, or drugs mentioned in this manuscript.

Short Running Head

Histology of Mechanically Processed Adipose Tissue

Supplementary Materials

Supplementary Figure S1

The following heatmap illustrates the criteria of the OHAT Score in the single categories that were evaluated.

	Selection Bias		Confounding Bias			Attrition/Exclusion Bias			Selective Reporting Bias	Other Bias
	Item One	Item Two	Item One	Item Two	Item Three	Item One	Item Two	Item Three	Item One	Item One
**Cicione et al.**	2	2	2	2	2	2	2	2	2	2
**Eigenberger et al.**	2	2	2	2	-1	2	2	2	2	2
**Fan et al.**	2	2	2	2	-1	2	1	1	2	2
**He et al.**	2	2	-1	2	-1	2	2	2	2	2
**Ragni et al.**	2	2	-1	2	-1	2	2	2	2	2
**Säljö et al.**	2	-1	2	2	-1	2	2	2	2	-1
**Sesé et al.**	2	2	2	2	-1	2	2	2	2	2
**Tran et al.**	2	2	2	2	-1	2	2	2	2	2
**Van Dongen et al.**	2	2	-1	2	-1	2	2	2	2	2
**Wu et al.**	2	2	-1	2	-1	2	2	2	2	2
**Yao et al.**	2	1	-1	2	-1	2	1	1	2	2
**Yu et al.**	2	2	2	2	2	2	2	2	2	2
**Zhang et al.**	2	2	2	2	-1	2	2	2	2	2
**Zhao et al.**	2	-1	-1	2	-1	2	2	1	2	2
**Zhu et al.**	2	2	2	2	-1	2	2	2	2	2

The evaluated categories were:

Selection Bias:

Item one: Was allocation to study groups adequately concealed?

Item two: Did selection of study participants result in appropriate comparison groups?

Confounding Bias:

Item one: Did the study design or analysis account for important confounding and modifying variables?

Item two: Were experimental conditions identical across study groups?

Item three: Were the research personnel and human subjects blinded to the study group during the study?

Attrition/Exclusion Bias:

Item one: Were outcome data complete without attrition or exclusion from analysis?

Item two: Can we be confident in the exposure characterization?

Item three: Can we be confident in the outcome assessment?

Selective Reporting Bias:

Item one: Were all measured outcomes reported?

Other Bias:

Were there no other potential threats to internal validity (e.g., statistical methods were appropriate and researchers adhered to the study protocol)?

As stated in the main text, Each domain was rated as: "++" (definitely low risk of bias), "+" (probably low risk), "−" (probably high risk), or "−−" (definitely high risk). Based on these ratings, studies were categorized into three levels: Level 1 (low risk of bias), if a maximum of one domain received a "−" rating and none received "−−"; Level 2 (moderate risk), if up to three domains were rated "−" and none "−−"; and Level 3 (high risk), if at least one domain was rated "−−" or more than three domains were rated "−".
